# The Role of Probiotics in Colorectal Cancer Management

**DOI:** 10.1155/2020/3535982

**Published:** 2020-02-14

**Authors:** Bhagavathi Sundaram Sivamaruthi, Periyanaina Kesika, Chaiyavat Chaiyasut

**Affiliations:** Innovation Center for Holistic Health, Nutraceuticals, and Cosmeceuticals, Faculty of Pharmacy, Chiang Mai University, Chiang Mai 50200, Thailand

## Abstract

Colorectal cancer (CRC) is one of the most common cancerous diseases worldwide and causes leading cancer-associated deaths. Several factors are related to the incidence of CRC such as unhealthy diet and lifestyle, heredity, metabolic disorders, and genetic factors. Even though several advanced medical procedures are available for CRC treatment, the survival rates are poor with many adverse treatments associated side effects, which affects the quality of life. Probiotics are a well-known bioactive candidate for the treatment of several diseases and ill-health conditions. The recent scientific evidence suggested that probiotic supplementation protects the CRC patients from treatment-associated adverse effects. The manuscript summarizes the influence of probiotic supplementation on the health status of CRC patients and discusses the possible mechanism behind the protective effect of probiotics against CRC. The literature survey revealed that beneficial impact of probiotic supplementation depends on several factors such as strain, dosage, duration of the intervention, host physiology, and other food supplements. The probiotic intervention improves the microbiota, releases antimicrobials and anticarcinogenic agents, helps to remove carcinogens, and improves the intestinal permeability, tight junction function, and enzyme activity in CRC patients. Besides, not all probiotic strains exhibit anti-CRC activities; it is necessary to screen the potent strain for the development of a probiotic-based therapeutic agent to control or prevent the incidence of CRC.

## 1. Introduction

Colorectal cancer (CRC) is one of the most common (∼1.4 million cases of CRC in 2012) cancerous disease worldwide and cause leading cancer-associated deaths (∼700 thousands of mortality) [1]. Several factors are associated with the incidence of CRC such as unhealthy diet and lifestyle, heredity, metabolic disorders, and genetic factors [2–5]. Indeed, 70% of the CRC incidents are related to environmental factors, and it has increased in technologically developed countries due to lack of physical activities [6, 7]. The gut microbiota is closely associated with the incidence and development of CRC [8]. The altered gut microbiota can provoke the carcinogenesis by altering the immune response, epithelial hemostasis, metabolic profile and activity, DNA damage, and irregular cellular and molecular activities in colonocytes [8–11].

Even though several advanced medical procedures (chemotherapy, surgery, immune and radiation therapy) are available for CRC treatment, the survival rates are poor with many adverse treatment-associated side effects, which affects the quality of the life [8]. Probiotics are a well-known bioactive candidate for the treatment of several diseases, and ill-health conditions [12–18]. The administration of probiotics in an adequate amount confers the health benefits to the host by positive regulation of the gut microbiota. Dysregulation of the microbiota is one of the major factors of development of CRC. The studies suggested that the intervention of probiotics protects the CRC patients from treatment-associated adverse effects compared to the respective control populations in the studies [19–21].

The competition for adhesion site, production of microbicidal agents such as bacteriocin, improvement of intestinal permeability, release of bioactive metabolites, regulation of immune pathways, and stimulation of cell protective responses are the key functions of a potent probiotic strain, thereby aiding to prevent the tumorigenesis, not limited to, of CRC [8].

In this review, the authors discussed the influence of probiotic supplementation on the health status of CRC patients and highlighted the results of *in vitro* and *in vivo* studies related to CRC and probiotics. They also discussed the possible molecular mechanism behind the health-promoting property of probiotics against CRC.

The literature was collected from Scopus, PubMed, Google Scholar, and ResearchGate using the search terms “probiotics” and “colorectal cancer”. The scientific documents (*n* = 50) were selected based on the information relevant to the scope of the current manuscript without any chronological restrictions.

## 2. Evidences of Anti-CRC Activities of Probiotics

### 2.1. In Vitro Studies

Baldwin et al. [22] demonstrated the effect of live or inactive probiotic strains (different concentrations of *Lactobacillus acidophilus*, and *L. casei*; total CFU are 1 × 10^6^, 1 × 10^7^, 1 × 10^8^, and 1 × 10^9^ CFU per ml) on the apoptotic capacity of 5-fluorouracil (5-FU) in the colorectal cancer cell line (LS513). The cotreatment of live or inactive *L. acidophilus*, *L. casei* (total probiotic concentration 1 × 10^8^ CFU/ml), and 5-FU (100 *µ*g/ml) enhanced the apoptotic efficiency (40%) of 5-FU in LS513 cells. The bacterial strains were inactivated through *γ* irradiation or through microwave radiation. Irradiation-mediated inactivated probiotic strains also enhanced the apoptotic activity of 5-FU similar to the enhancing level of live probiotic strains at all concentrations. But microwave-treated probiotic strains reduced the apoptotic activity of 5-FU. Probiotic-mediated enhancement of apoptotic activity of 5-FU was dose-dependent. Probiotic strains (1 × 10^8^ CFU per mL) and 5-FU exposure induced the caspase-3 activation and reduced the p21 expression faster in LS513 cells. The results suggested that use of potent probiotic strains can improve the efficacy of 5-FU [22].

Escamilla et al. [23] studied the effect of cell-free supernatants (CFS) from *L. casei* and *L. rhamnosus* GG on the invasion of human colorectal cancer cell line (HCT-116). CFS from both probiotic strains significantly reduced the HCT-116 cell invasion. CFS exposure reduced the matrix metalloproteinase-9 level and increased the zona occludens-1 level in HCT-116 cells. The inhibitory activities were not observed when HCT-116 cells were treated with CFS from commensal bacteria *Bacteroides thetaiotaomicron*. The active compounds were found to be present in the 50–100 kDa and >100 kDa fractions of CFS from both *Lactobacillus* strains. The study proved that secretory metabolites of *L. casei* and *L. rhamnosus* GG have anti-invasive activity in HCT-116 cells [23].

Orlando et al. [24] assessed the effect of live or heat-killed cells of *L. paracasei* IMPC2.1 and *L. rhamnosus* GG (10^8^ CFU/mL) on the proliferation and apoptosis of gastric (HGC-27) and colon (DLD-1) cancer cell lines. Both live and heat-killed cells (*L. paracasei* IMPC2.1 and *L. rhamnosus* GG) effectively reduced the proliferation and induced the proapoptosis in both cancer cells *in vitro*. Hence, the cells of IMPC2.1 (heat-killed) can be used for the preparation probiotic-based functional food to improve the health status of CRC patients as a complementary regimen [24].

Soltan Dallal et al. [25] investigated the effect of CFS and bacterial extracts of probiotic strains (*L. acidophilus* ATCC 4356 and *L. casei* ATCC 39392) on the proliferation and apoptosis of colorectal cancer cell line (CaCo-2). Both CFS and bacterial extract of *L. acidophilus* ATCC 4356 and *L. casei* ATCC 39392 effectively reduced the proliferation, migration, and invasion of CaCo-2 cells and induced the apoptosis while cell necrosis was not induced by CFS treatment, whereas bacterial extract induced the cellular necrosis in CaCo-2 cells. The study suggested that CFS and bacterial extract of *Lactobacillus* strain impeded the malignant phenotype of CaCo-2 cells [25].

An and Ha [26] studied the effect of *L. plantarum* CFS on the characteristics of 5-FU-resistant HT-29 and HCT-116 cells. They also examined the effect of *L. plantarum* CFS on the therapeutic capacity of 5-FU in 5-FU-resistant HT-29 and HCT-116 cells. Exposure (72 h) of *L. plantarum* CFS (10 *μ*g) significantly reduced the expression of CD44, CD133, CD166, and ALDH1 in 5-FU-resistant HT-29 and HCT-116 cells. The combinational treatment of *L. plantarum* CFS (10 *μ*g) and 5-FU (50 *μ*M) hindered the Wnt/*β*-catenin signaling and also increased the activity of caspase-3 and suppressed the formation and size of colonospheres in 5-FU-resistant HT-29 and HCT-116 cells. CFS from *L. plantarum* enhanced the therapeutic capacity of 5-FU in 5-FU-resistant colorectal cancer cells [26]. Cousin et al. [27] investigated the synergistic effect of *Propionibacterium freudenreichii* ITG P9 and TRAIL (tumor necrosis factor-related apoptosis-inducing ligand) in HT-29 cells. Combination of *Propionibacterium freudenreichii* ITG P9 (CFS or metabolites) and TRAIL (TNF-related apoptosis-inducing ligand) treatment synergistically induced proapoptosis and suppressed the antiapoptotic gene expression in HT-29 cells. The proapoptotic activity of combination therapy was dependent on death receptors TRAIL-R1/DR4, TRAIL-R2/DR5, and caspase activity. ITG P9-mediated fermented milk also exhibited the same apoptosis-inducing activity in combination with TRAIL indicating that CFS and metabolites of probiotics (ITG P9), and ITG P9-mediated fermented milk increased the efficacy of chemotherapy in CRC cells [27].

Chen et al. [28] evaluated anti-CRC property of *Lactobacillus* strains (*L. brevis* PM150, *L. plantarum* PM153, *L. brevis* PM177, *L. delbrueckii* subsp. *bulgarius* BCRC10696, *L. reu*teri BCRC14625, *L. salivarius* BCRC14759, and *L. johnsonii* BCRC17010) in HT-29 cells. The study revealed that *L. johnsonii* BCRC17010 was a potent probiotic strain with high adhesion property and induced proapoptotic process and lactate dehydrogenase release in HT-29 cells and effectively inhibited the growth of HT-29 cells [28].

Kahouli et al. [29] demonstrated the effect of probiotic mix (*L. acidophilus* ATCC 314 and *L. fermentum* NCIMB 5221) on CaCo-2 cells. Probiotic mix treatment significantly reduced the proliferation of cancer cells and induced the apoptosis in CaCo-2 cells [29].

Saber et al. [30] examined the effect of secretion metabolites of *Pichia kudriavzevii* AS-12 on HT-29 and Caco-2 cells. Methanolic extract of secreted metabolites of *Pichia kudriavzevii* AS-12 (MEPK) was cytotoxic to HT-29 and Caco-2 cells, and the cytotoxic effect in HT-29 cells was comparable with that of 5-FU. The expression of BAD, caspase-3, caspase-8, caspase-9, and Fas-R was increased, while Bcl-2 expression was suppressed in MEPK-treated cancer cells. The level of proapoptotic genes (caspase-3, caspase-9, Fas-R in HT-29 cells, and Fas-R in Caco-2 cells) expression was higher in MEPK treated cells than 5-FU treated cells (positive control). Based on the results, the MEPK can be considered as a potent anticancer agent [30].

Sambrani et al. [31] studied the effect of *Saccharomyces cerevisiae* on the apoptosis, metastasis, and growth of HT-29 cells. CFS from *S. cerevisiae* exhibited antiproliferative activity in HT-29 cells by suppressing the expression of *Bclxl* and *RelA* and inducing the expression of *PTEN* and *Caspas3* at 24 h posttreatment [31]. Gong et al. [32] evaluated the effect of *L. acidophilus* HB56003, *Streptococcus thermophilus* HB5621, *Enterococcus faecalis* HB62001, and *Bifidobacterium longum* HB55020 on human colonic smooth muscle strips. CFS, cellular fractions, and live cells of *L. acidophilus* HB56003, *S. thermophilus* HB5621, *E. faecalis* HB62001, *B. longum* HB55020 significantly inhibited the contractility of human colonic smooth muscle strips *in vitro* condition. NG-nitro-L-arginine (an inhibitor of nitric oxide synthase) treatment reduced the inhibitory property of CFS from HB5621 and HB62001, but inhibitory property of HB55020 and HB56003 was not affected. The inhibitory activities of probiotics were dose-dependent [32] (Table 1).

### 2.2. In Vivo Studies

Le Leu et al. [33] examined the effect of the intervention of probiotic (*B. lactis*; 1 × 10^11^ CFU/g) or prebiotic (Hi-maize® 958 or Hi-maize® 260; high-amylose maize starch was used as a source of resistant starch 100 g/kg diet) or synbiotic (*B. lactis* + resistant starch) on incidence and development of colon neoplasm in azoxymethane-mediated colonic neoplasm-induced Sprague Dawley rats. About 22 weeks of intervention showed that synbiotic preparation significantly reduced the incidence and proliferation of colon neoplasm. The changes in short-chain fatty acid content and variations in pH were also observed in the prebiotic group. Probiotic intervention exhibited no protective effects against CRC in the rat model. The study clearly revealed that the synbiotic intervention is a better protective agent compared to pre- or probiotic regimens [33].

Appleyard et al. [34] investigated the effect of probiotic (VSL#3) supplementation on colitis-associated CRC. The intervention of VSL#3 (a mixture of eight probiotic strains such as *B. breve, B. infantis, B. longum, L. acidophilus, L. bulgaricus, L. casei, L. plantarum*, and *Streptococcus salivarius* subsp. *thermophilus*) at the concentration of 5 × 10^9^ CFU/100 g of body weight to trinitrobenzene sulfonic acid-mediated chronic colitis-induced Sprague Dawley rats significantly prevented the development of carcinoma, the incidence of high-grade dysplasia, and colon damages compared to control. The probiotic supplementation increased the expression of angiostatin, alkaline sphingomyelinase, and vitamin D receptor in experimental rats. The results suggested that VSL#3 protects the experimental rats from CRC development by diminishing the inflammatory responses and delay the progress of dysplasia [34]. Do et al. [35] studied the effect of probiotic (VSL#3) supplementation on colitis-associated CRC. VSL#3 (1.3 × 10^6^ CFU/day) along with anti-inflammatory agent balsalazide (300 mg/kg body weight/day) effectively protected the experimental mouse from azoxymethane/dextran sodium sulfate-induced colitis-associated carcinogenesis. The supplementation of VSL#3 and balsalazide reduced the expression of p-STAT3 (phospho-signal transducer and activator of transcription 3) and BCL-2 (B-cell lymphoma 2) and decreased the level of MIP-1*β* (macrophage inflammatory protein 1 beta), MCP-1 (monocyte chemoattractant protein-1), IL-6 (interleukin-6), IL-10, and number of F4/80-positive macrophages and increased the BAX (BCL2-associated X protein) expression in CRC mice. The results suggested that the combination of VSL#3 and balsalazide could be an adjuvant therapeutic agent for CRC [35]. Another study revealed that the supplementation of VSL#3 was not interfering the azoxymethane-induced colitis-associated CRC development in *Il10*^*-/-*^ mouse model but altered the mucosal-adherent microbiota [36].

Verma and Shukla [37] examined the effect of supplementation of *L. rhamnosus GG,* or *L. casei,* or *L. plantarum,* or *L. acidophilus*, or *B. bifidum* (probiotic dose: 1 × 10^9^ CFU/day) for seven weeks (1 week before starting 1,2-dimethylhydrazine exposure and continued for six weeks) on the 1,2-dimethylhydrazine- (DMH-) induced colon carcinogenesis in Sprague Dawley rats. Supplementation of *L. rhamnosus GG* or *L. acidophilus* effectively reduced the aberrant crypt foci (ACF) formation and *β*-glucuronidase activity in DMH-mediated CRC induced rats. Supplementation of *L. plantarum* or *L. casei,* reduced the nitroreductase, and supplementation of *B. bifidum* reduced *β*-glucosidase activities in DMH-mediated CRC-induced rats. The morphological changes were hindered in the DMH-mediated CRC-induced rats of probiotic-supplemented group compared to nonprobiotic group. The results suggested that *L. rhamnosus GG* and *L. acidophilus* exhibited better anti-CRC activities in DMH-mediated CRC-induced rats [37]. The further extended study revealed that the supplementation of synbiotic preparation consists of *L. rhamnosus GG, L. acidophilus,* and inulin displayed superior prophylactic activity by enhancing the antioxidant system in DMH-mediated CRC-induced rats compared to that of the supplementation of probiotic or prebiotic [38].

Mohania et al. [39] studied the effect of probiotics with or without piroxicam on DMH-induced colon carcinogenesis in male Wistar rats. The supplementation of *L. acidophilus* LaVK2 + *B. bifidum* BbVK3 and piroxicam significantly reduced the DMH-induced preneoplastic lesions (ACF, mucin-depleted foci) in rats. The ratio of aberrant crypts and ACF, large mucin-depleted foci, and proliferating cell nuclear antigen were also significantly decreased by probiotic supplementation. The results suggested that supplementation of probiotics along with piroxicam exhibits better protective activity in DMH-mediated CRC-induced rats [39].

Kumar et al. [40] investigated the effect of *L. plantarum* AS1 on DMH-induced colon carcinogenesis in male Wistar rats. The pre- or post- or both (pre- and post-) supplementation of *L. plantarum* AS1 (10^9^ CFU/day) for 5–21 weeks significantly improved the antioxidant status of DMH-induced CRC rat and positively altered the lipid peroxidation and selected biomarkers (superoxide dismutase, catalase, glutathione S-transferases, alkaline phosphatase, and acid phosphatase). The number and diameter of the tumor and the histopathological scores were reduced in AS1 supplemented group compared to control. The results suggested that AS1 supplementation, both pre- and postsupplementation, protects the DMH-induced CRC in the rat by enhancing the antioxidant system of the host [40].

Zhu et al. [41] investigated the effect of *L. salivarius* on DMH-induced colon carcinogenesis in male F344 rats. *L. salivarius* (5 × 10^8^ or 1 × 10^10^ CFU/Kg body weight/day for 15 weeks) supplementation improved the colonic microflora (reduced the *Bacillus* and *Ruminococcaceae* strains) and luminal metabolisms in DMH-mediated CRC-induced rats. The significant level of increase in short-chain fatty acids and a notable reduction in azoreductase activity was observed in the probiotic-treated group, while *β*-glucosidase and *β*-glucuronidase activities were not affected compared to control. The study suggested that the supplementation of *L. salivarius* positively altered the microbiota and enzyme activities in DMH-mediated CRC-induced rats [41].

Hu et al. [42] studied the effect of probiotic strains (*L. plantarum* or *L. rhamnosus*) in CT26 tumor-bearing BALB/c mice. BALB/c mice were presupplemented with *L. plantarum* or *L. rhamnosus* (1 × 10^9^ CFU/day) for 14 days and CT26 carcinoma cells were introduced to induce cancer in mice. The changes in immune regulations and status of tumor growth have been monitored in CT26 tumor-bearing mice. *L. plantarum* pre-exposure significantly reduced the CT26 cell growth and increased the lifespan of tumor-bearing mice by improving the Th1-type CD4+ T differentiation, NK cell infiltration, CD8^+^ function, and IFN-*γ* expression compared to *L. rhamnosus* pre-exposed group and control. The results proved that *L. plantarum* exhibited antitumor immune-enhancing property [42].

Zhang et al. [43] investigated the effect of *L. salivarius* Ren on DMH-induced colon carcinogenesis in male F344 rats. The supplementation of *L. salivarius* Ren (5 × 10^10^ CFU/Kg body weight/day) for 32 weeks reversed the DMH-induced altered microbiota in experimental rats. The level of *Clostridiales*, *Bacteroides dorei*, and *Ruminococcus* species have been reduced, and the amount of *Prevotella* species increased in *L. salivarius* Ren supplemented group. The results suggested that *L. salivarius* Ren supplementation protects the experimental animals from DMH-induced CRC via positive regulation of microbiota [43].

Gamallat et al. [44] investigated the effect of *L. rhamnosus* GG on DMH-induced colon carcinogenesis in Sprague Dawley rats. *L. rhamnosus* GG CGMCC 1.2134 (1 × 10^9^ CFU/day) intervention for 25 weeks significantly reduced the incidence, multiplicity, and volume of the tumor in DMH-induced CRC rat model. Also, the expression of TNF-*α*, COX-2, NFkB-p65, Bcl-2, and *β*-catenin were reduced, and Bax, p53, and casp3 expressions were increased in the probiotic-supplemented group compared to the nonprobiotic group. The results indicated that *L. rhamnosus* GG CGMCC 1.2134 could diminish the CRC-associated inflammatory reactions, thereby protecting the host system [44].

Lenoir et al. [45] investigated the effect of *L. casei* BL23 on DMH-induced colon carcinogenesis in C57BL/6 mice. The C57BL/6 mice were presupplemented with 10 *µ*l (1 × 10^8^ CFU/*µ*l) of *L. casei* BL23 on day (0, 14, and 28), and then CRC induction was started on the 35^th^ day and continued weekly during 10 weeks. Presupplementation of *L. casei* BL23 significantly protected the mice from CRC via altering the regulation of *T*_reg_ and Th17 T-cell-associated cytokines. Particularly, *L. casei* BL23 supplementation reduced the incidence of the tumor and the number of multiple plaque lesions and improved the histopathological score. The expression of IL-6, IL-10, IL-17, and TGF-*β* and the ratio of IL-10/TNF-*α* were increased in the probiotic-treated group. Collectively, the results suggested that *L. casei* BL23 protects the mice from DMH-induced CRC via *T*_reg_ and Th17 T-cell regulation [45].

Mi et al. [46] assessed the chemoprotective effect and anti-CRC property of *B. infantis* in DMH and SW480 cell induced CRC rat model. The CRC induced animals were supplemented with *B. infantis* (1 × 10^9^ CFU/day) and/or 5-FU + Oxaliplatin for 11 days, and the animals were examined for several pathological assessments. The level of IL-6, IL-1*β*, TNF-*α* levels, and Th17 and Th1 cells-associated cytokines were reduced and the level of CD4^+^, CD25^+^, Foxp^3+^, and *T*_regs_ were increased in probiotic-supplemented CRC rat. The results collectively showed that the supplementation of probiotic effectively reduced the chemotherapy-associated health damages in a rat model [46].

Kahouli et al. [29] demonstrated the effect of probiotic mix (*L. acidophilus* ATCC 314 and *L. fermentum* NCIMB 5221) on Apc^Min/+^ CRC mouse model. The supplementation of probiotic formula consists of 0.5 × 10^10^ CFU of *L. acidophilus* ATCC 314, and 0.5 × 10^10^ CFU of *L. fermentum* per day for 12 weeks reduced the severity of CRC in Apc^Min/+^ CRC mouse model. The number and multiplicity of the tumor and expression of cellular proliferation markers were reduced significantly in the probiotic-treated group compared to control. The results claimed that the prepared probiotic regimen could be used as a biotherapeutic agent to avert the CRC [29].

Song et al. [47] studied the effect of probiotic Bifico on azoxymethane/dextran sodium sulfate-induced colitis-associated carcinogenesis in C57BL/6 mice. The supplementation of probiotic mix (*B. longum, L. acidophilus*, and *E. faecalis*; 1.2 × 10^7^ CFU/day for 2 weeks pretreatment and continued till the end of the experiment) significantly reduced the tumor formation and intestinal inflammation, and improved the diversity and abundance of microbiota and altered the expression of CXCR2 ligand genes in azoxymethane/dextran sodium sulfate-induced colitis-associated cancer mice model [47].

Heydarii et al. [48] investigated the influence of supplementation of probiotics on the microRNA regulation in azoxymethane-induced CRC mice. About 5-month intervention of *L. acidophilus* (1 × 10^9^ CFU/day) and *B. bifidum* (1 × 10^9^ CFU/day) improved the expression pattern of miRNA-associated with cancer prevention. The relative expression of miR-135b, miR-155, and KRAS have been reduced while the expression of miR-26b, miR-18a, APC, PU.1, and PTEN were increased in the probiotic-treated group compared to nonprobiotic group. The study suggested that the probiotic exhibited antitumor property by regulating the miRNAs and associated genes in CRC experimental mice [48].

Lin et al. [49] revealed that the supplementation of *L. acidophilus* LA5 and/or *B. animalis* subsp. *lactis* BB-12 (5 × 10^7^ CFU of single probiotic strain/day or 2.5 × 10^7^ CFU each strain/day) and germinated brown rice extract (GBR; 10% in diet) for 10 weeks reduced the mucin-depleted foci formation, ACF-producing sialomucin and expression of anti-apoptotic Bcl-2 in azoxymethane/dextran sodium sulfate-induced CRC rat model. The supplementation of probiotics and GBR protects the CRC rat by increasing the expression of p53, Bax, caspase-3, and Bax/Bcl-2 ratio and restored the SOD activity. The results suggested that GBR and probiotic supplementation improved the antioxidant machinery of the host system and induced the apoptosis in tumor cells [49].

Sharaf et al. [50] studied the protective effect of probiotic strains (*L. rhamnosus* GG MTCC #1408 and/or *L. acidophilus* NCDC #15) along with anti-inflammatory drug (celecoxib) in DMH-induced CRC rats. Supplementation of 1 × 10^9^ CFU/day of probiotics (single strain or multistrain) and/or celecoxib (6 mg/kg body weight) for 18 weeks significantly reduced the tumor burden and multiplicity of the tumor. The expression studies suggested that studied regimen effectively reduced the expression of antiapoptotic genes (Bcl-2, K-ras) and increased the tumor suppressor and proapoptotic genes (p53 and Bax) in the CRC rat model. The study suggested that the combination of multistrain probiotic preparation and celecoxib exhibited superior protective activity compared to single-strain regimen [50]. Another study showed that even 6 weeks of the intervention of probiotics, especially *L. rhamnosus* GG, and celecoxib significantly reduced the *β*-catenin, COX-2, and NF-*κ*B expression, and formation of ACF in DMH-induced CRC rats [51] (Table 2).

## 3. Clinical Trials

Österlund et al. [52] conducted a randomized, phase III, open-label, 2 × 3 factorial design study to investigate the influence of probiotic supplementation on chemotherapy-induced diarrhea in CRC patients (individuals with Dukes' stage B CRC or Dukes' stage C CRC or Dukes' stage D CRC who have undergone surgery). The patients received 5-FU-based postoperative adjuvant chemotherapy (at Helsinki University Central Hospital, Finland) for 24 weeks, and they were supplemented with probiotic strain *L. rhamnosus* GG (1–2 × 10^10^/day) capsules and guar gum fiber containing nutritional supplement during the adjuvant chemotherapy. The probiotic supplementation significantly reduced the frequency of diarrhea and abdominal discomfort. The results suggested that *L. rhamnosus* GG can be used as an adjuvant therapy to diminish chemotherapy-associated diarrhea and gastrointestinal discomfort [52]. Golkhalkhali et al. [53] conducted a double blind, randomized controlled trial with CRC patients to study the effect of strain-specific probiotic mix and *ω*-3 fatty acid on XELOX chemotherapy (at University of Malaya Medical Centre, Malaysia). The supplementation of probiotic preparation (a mixture of *L. casei, L. acidophilus*, *L. lactis, B. bifidum*, *B. longum*, and *B. infantis* strains; 30 × 10^9^ CFU/sachet; 2 sachets per day for 4 weeks), and *ω*-3 fatty acid (2 g per day for 8 weeks) improved the health condition in CRC patients. The chemotherapy-associated inflammatory reactions were significantly nullified in the treatment group supplemented with probiotic and *ω*-3 fatty acid. The level of IL-6 was reduced in the treatment group compared to the placebo group. The study suggested that probiotic intervention reduced the chemotherapy-induced inflammatory dysregulation and improved the quality of life in CRC patients [53].

Rafter et al. [54] studied the effect of dietary synbiotic on the risk of CRC in polypectomized (*n* = 43) and CRC (*n* = 37; 6 individuals with Dukes' stage A CRC, 17 individuals with Dukes' stage B CRC, and 14 individuals with Dukes' stage C CRC) patients. The supplementation of synbiotic formula (*L. rhamnosus* GG, *B. lactis* Bb12, and oligofructose-enriched inulin) significantly reduced tumor proliferation and improved the barrier function in polypectomized patients. Moreover, the intervention of synbiotic formula regulated the microbiota, a notable level of increase in *Lactobacillus* and *Bifidobacterium* species, and a decrease in *Clostridium perfringens* have been observed in the fecal samples of the patients. The release of IFN-*γ* and IL-2 were also altered after synbiotic supplementation in CRC patients. The results suggested that the supplementation of synbiotic preparation beneficially altered the CRC-associated biomarkers [54].

Gao et al. [55] investigated the effect of probiotic mix on CRC patients (individuals with CRC who have undergone radial colorectomy at Sixth People's Hospital affiliated Shanghai Jiao Tong University, Shanghai, China). The intervention of probiotic regimen (*B. longum, L. acidophilus,* and *E.* faecalis; 6 × 10^7^ CFU/day) for five days significantly improved diversity and density of mucosa-associated microbiota and decreased the *Fusobacterium* species in CRC patients. The results suggested that probiotic can improve the health status of CRC patients via positive regulation of mucosal-associate microbiota [55].

Ishikawa et al. [56] studied the protective effect of dietary fiber and probiotics by conducting a randomized clinical trial with the human volunteers (who had colorectal tumors removal surgery). The volunteers were supplemented with *L. casei* strain Shirota (*n* = 96) or wheat bran (*n* = 95) or both (*n* = 96), and volunteers with no treatment (*n* = 93) for four years. The probiotic-supplemented group showed a significant reduction in the incidence of tumor formation compared to wheat bran supplemented group and control group after four years. Atypia of colorectal tumors has been significantly prevented by the probiotic treatment [56].

Worthley et al. [57] conducted a placebo-controlled double blind crossover trial to study the effect of supplementation of prebiotic, probiotic, and synbiotic preparation on the biomarkers of CRC in healthy human volunteers. All the volunteers were supplemented with probiotics (*B. lactis*; 1 × 10^9^ CFU/g; 5 g/day), prebiotics (resistant starch; 25 g/day), and synbiotics in a sequential way, and each intervention lasts for 4 weeks with no washout period. Changes in the microbiota, DNA methylation, epithelial proliferation, and biomarkers of CRC has been assessed after 4 weeks of each intervention. The results suggested that the supplementation of synbiotic preparation effectively altered the microbiota than other interventions. Moreover, synbiotic supplementation did not significantly affect the serum, fecal, and epithelial biomarkers [57].

Gianotti et al. [58] conducted a double-blind randomized controlled trial to study the effect of probiotics (*B. longum* and *L. johnsonii*; low dose 2 × 10^7^ CFU per day or high dose 2 × 10^9^ CFU per day) when supplemented perioperative to CRC patients (individuals with CRC who are undergoing elective colorectal surgery). The perioperative (pre-, on day, and postsurgery) supplementation of probiotics (high dose of *B. longum* and *L. johnsonii*) to CRC patients significantly reduced the members of *Enterobacteriaceae* in fecal samples. *L. johnsonii* was observed in the stool samples or in biopsy samples of CRC patients supplemented with probiotics. But presence of *B. longum* was not observed in the stool samples or in biopsy samples of CRC patients in probiotic group. Adherence of *L. johnsonii* was directly correlated with the probiotic dose. The expression of CD3, CD4, CD8, and lymphocyte subsets was increased, and the dendritic cells were not affected and activated, significantly. All the observed changes were directly correlated with the dose of the probiotic supplementation. The study suggested that *L. johnsonii* can improve the health status of CRC patients by adhering the colonic mucosa and altering the microbiota and immune system [58]. Liu et al. [59] estimated the effect of perioperative probiotic supplementation on the gut barrier function and postsurgery-related infectious complication in CRC patients (individuals with CRC who are undergoing elective colorectal surgery). The pre- and postsurgery supplementation of probiotic preparation (*L. plantarum, L. acidophilus,* and *B. longum*; 2.6 × 10^14^ CFU/day) significantly reduced the permeability of horseradish peroxidase, bacterial translocation, lactulose⁄mannitol ratio, enteropathogenic bacterial load, and incidence of postoperational diarrhea and infections and improved the transepithelial resistance and expression of tight junction protein in CRC patients [59]. Liu et al. [60] investigated the effect of perioperative probiotic supplementation on the serum zonulin level and postsurgery-related infectious complication in CRC patients (individuals with Dukes' stage A CRC, Dukes' stage B CRC, or Dukes' stage C CRC who are undergoing colorectal surgery). The supplementation of same probiotic formula [59] significantly reduced the serum zonulin level, duration of the postoperative antibiotic treatment, pyrexia, and infection in CRC patients. The p38 mitogen-activated protein kinase pathway was also hindered during probiotic supplementation. The results suggested that the probiotic formulation comprises *L. plantarum, L. acidophilus,* and *B. longum* improved the serum zonulin level and postsurgery-related infectious complications in CRC patients [60].

Hibberd et al. [61] studied the effect of probiotics on the microbiota in CRC patients (individuals with CRC stage I–III). The CRC patients (*n* = 8) were supplemented with probiotics (ProBion Clinica, 2 tablets containing 1.4 × 10^10^ CFU of *B. lactis*, 7 × 10^9^ CFU of *L. acidophilus*, and 630 mg inulin per day) for 8–78 days until the day of surgery (intervention duration varied depends on the duration between the diagnosis to surgery period). The biopsy samples (both tumor and normal mucosa) were collected from CRC patients of probiotics group, and CRC patients (*n* = 7) of nonprobiotic group during both colonoscopy and surgery. Normal mucosal biopsies were also collected from noncancer control groups (*n* = 21; individuals with normal colonic mucosa) during colonoscopy. Fecal samples were obtained from all participants (CRC patients and noncancer individuals) after colonoscopy and from CRC patients at surgery. The results showed that the microbiota of tumor-associated samples was enriched with tumor-related microbial niche compared to control subjects. Probiotic intervention improved the diversity and abundance of butyrate-producing bacteria (*Clostridiales* and *Faecalibacterium* species) in the fecal and mucosal (tumor and normal mucosa) microbiota of CRC patients. Probiotic intervention also reduced the level of *Fusobacterium* and *Peptostreptococcus* species (which are considered as tumor-inducing microbial agents) in fecal microbiota of CRC patients [61].

Lee et al. [62] investigated the effect of probiotic (*L. rhamnosus* R0011 and *L. acidophilus* R0052; 2 × 10^9^ CFU/tablet, twice a day for 12 weeks) on the quality of life in CRC survivors (individuals who have completed the treatment between 6 weeks and 2 years before the study) by conducting a randomized, double-blind placebo-controlled trial. The quality of life improvement was assessed by questionnaires. The results suggested that the supplementation of probiotic formula improved the health-span (improvement in irritable bowel symptoms, CRC-related health issues, functional well-being scores) of the participants significantly [62].

Aisu et al. [63] studied the effect of perioperative probiotic supplementation on the postsurgery-related infectious complication in CRC patients (individuals with CRC stages I, II, IIIA, IIIB, and IV who are undergoing elective colorectal surgery). The supplementation of BIO-THREE® (*Enterococcus faecalis* T110, *Clostridium butyricum* TO-A, and *Bacillus mesentericus* TO-A) to perioperative CRC patients for 3–15 days (before surgery) significantly reduced the postoperational superficial incisional surgical site infections compared to nonprobiotic group and also improved the microbiota and immune system positively [63]. Tan et al. [64] examined the effect of perioperative probiotic (HEXBIO®) supplementation in promoting the recovery and returning to normal gut function in CRC patients (individuals with CRC stages I, II, III, and IV who are undergoing elective colorectal surgery). The perioperative supplementation of HEXBIO® (a mixture of *L. acidophilus, L. casei, L. lactis, B. infantis. B. bifidum,* and *B. longum*; 30 × 10^9^ CFU/sachet; twice per day) to CRC patients for seven days (prior to surgery) significantly reduced the time required for regaining normal gut function after surgery and also reduced the duration of the hospital stay compared to the placebo group. The study suggested that the perioperative supplementation probiotic formulations could help to improve the health status of CRC patients after surgery [64]. Yang et al. [65] studied the effect of postoperative probiotic supplementation on quality of life in CRC patients (individuals with sporadic CRC stages 0, I, II, III, who are undergoing confined colorectal resection surgery). The intervention of a combination of *B. longum*, *L. acidophilus*, and *E. faecalis* (each 1 × 10^7^ CFU per gram) for 12 days (5 days before surgery and 7 days after surgery) improved the bowel function and reduced the incidence of diarrhea in CRC patients compared to the placebo group [65].

He et al. [66] conduced a meta-analysis of randomized controlled trials to investigate the effect of perioperative probiotic or synbiotic supplementation in CRC patients (individuals with CRC who are undergoing colorectal resection surgery). The perioperative administration of probiotic or synbiotic regimen significantly reduced the incidence of diarrhea, pneumonia, and total infection in CRC patients. Additionally, probiotic or synbiotic supplementation improved the microbiota by increasing the *Lactobacillus* and reducing the *Enterobacteriaceae* members, but no significant changes were observed in length of hospital stay, incision and perineal infection, septic morbidity, and anastomotic leak [66]. Some of the recent meta-analysis studies revealed that the supplementation of probiotic preparations consists of *Lactobacillus* strains effectively reducing the surgical inflammation and promoting the surgical recovery in CRC patients [67], and the probiotic supplementation also effectively reduced the postoperative infection and complications such as incision infection, pneumonia, and flatus time [68] and also improved the intestinal mucosal barrier function in CRC patients [69].

Kotzampassi et al. [70] studied the effect of postoperative probiotic supplementation on the postsurgery-related infectious complication and quality of life in CRC patients (individuals with CRC who are undergoing colorectal surgery). The perioperative supplementation of LactoLevure® (*B. lactis*; 1.75 × 10^9^ CFU*, L. acidophilus*; 1.75 × 10^9^ CFU, *L. plantarum*; 0.5 × 10^9^ CFU, and *Saccharomyces boulardii*; 1.5 × 10^9^ CFU; twice per day) for 16 days (1 day before surgery, and 15 days after surgery) significantly reduced the postoperational complications compared to the placebo group. Specifically, the incidence of surgical site infection, anastomotic leakage, and pneumonia have been notably lower in the probiotic-supplemented group compared to placebo. Moreover, the expression of IL-6, TNF, and SOCS3 (suppressor of cytokine signaling 3) have been altered in a positive way to improve the quality of the postoperational life in CRC patients [70]. Theodoropoulos et al. [71] investigated the effect of postoperative synbiotic supplementation on the postsurgery-related infectious complication and quality of life in CRC patients (individuals with CRC stages 0, I, II, III, and IV, who are undergoing colorectal surgery). The supplementation of synbiotic preparation (Synbiotic Forte™, probiotics includes *Pediococcus pentosaceus*, *Leuconostoc mesenteroides, L. paracasei, L. plantarum*, and prebiotics such as inulin, pectin, *β*-glucan, and resistant starch) for 15 days considerably improved the Gastro-intestinal Quality of Life Index and functional bowel disorder score in CRC patients compared to the placebo group, while no changes were observed in “constipation” score. The study revealed that the supplementation of Synbiotic Forte™ improved the health condition of CRC patients after surgery, especially enhanced the gastrointestinal function [71] (Table 3).

### 3.1. A Possible Mechanism Underlying Anti-CRC Activity of Probiotics and Its Derivatives

**Figure 1:**
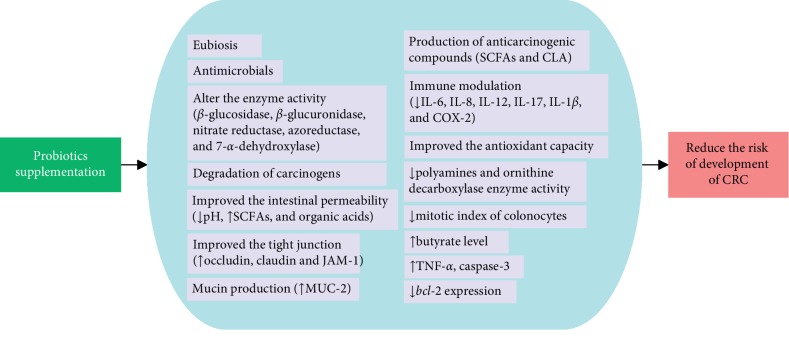
The possible mechanism underlaying the anticarcinogenic property of probiotics. CRC: colorectal cancer, SCFAs: short-chain fatty acids, CLA: conjugated linoleic acids, ↑: increased, and ↓: decreased.

Even though several studies attempted to explain the mechanism of the anticarcinogenic property of probiotics [72–75], a clear mechanism behind the anti-CRC activity of probiotic has not been described yet. Several evidences revealed that probiotics confer the health benefits by modifying the composition of microbiota and its metabolic activities, production of anticarcinogenic and antimicrobial compounds, improvement of antioxidant system of the host, degradation of carcinogens, alter the expression of inflammation-associated genes, immune enhancement, and prevention of cancerous proliferation and apoptotic induction (Figure 1).

Eventually, the continuous supplementation of any microbial preparation has an influence on the microbiota of the host. It has been proved that probiotic supplementation can positively alter the intestinal microbiota of the host system and aids to maintain the eubiosis [41, 43]. The probiotics can produce antimicrobial substances (like bacteriocins), which hinder the growth of pathogenic microbes in the intestinal lumen, thereby preventing the dysbiosis and development of CRC [76].

Some of the bacterial enzymes (produced by the members of *Clostridium*, *Bacteroides*, *Eubacterium*) such as nitrate reductase, azoreductase, *β*-glucosidase, *β*-glucuronidase, and 7-*α*-dehydroxylase are associated with the production of carcinogenic compounds such as cresols, ammonia, phenols, aglycones, and *N*-nitroso compounds, and these compounds induce the antiapoptotic pathways, thereby facilitating the development of CRC [77, 78]. Studies proved that the supplementation of probiotics reduced the activities of bacterial enzymes significantly [37, 39, 79, 80]. The carcinogenic compounds bind with peptidoglycan, present in the cell wall, of the probiotic microbes and excreted through feces. Some of the probiotic strain can metabolize the carcinogenic compounds especially amines and *N*-nitroso compounds [76], and the alternation of metabolic activity (i.e., reduced the endogenous production of carcinogenic compounds) of intestinal microbiota and binding and degradation of carcinogens are some of the mechanisms by which probiotic supplementation reduced the risk of development of CRC.

The compounds like short-chain fatty acids (SCFAs) such as butyrate, propionate, acetate, and conjugated linoleic acid (CLA) act as anticarcinogenic agents. Butyrate is a well-known SCFA associated with CRC. Lactic acid bacteria (LABs) do not produce butyrate but can convert the lactate and acetate into butyrate [76]. Most of the probiotic microbes are LABs. The supplementation probiotic will increase the concentration of SCFAs in the intestinal lumen that stimulated the release of antiinflammatory cytokines, suppressed the inflammatory pathways, and improved the antioxidant system [57, 81, 82]. Likely, CLA can induce the expression of PPAR-*γ*, which influence the immune system, lipid metabolism, and apoptosis process [72, 83].

The chronic inflammation is one of the lethal factors associated with the development of CRC, which disturb the intestinal microbiota [77, 84]. The healthy intestinal microbiota is crucial for the maturation of the immune system and development of immunity against invading pathogens. The supplementation of probiotics aids to improve the immune system and modulate the immune system via regulating the secretion of anti-inflammatory cytokines and associated regulatory genes [78, 85, 86].

The improvement of intestinal permeability is often associated with several gastrointestinal tract associated illness. The probiotic supplementation improves the gut barrier function [87]. The intestinal epithelial line is protected by three important factors such as pH, tight junction proteins, and secreted mucins. The metabolic activity of probiotics produces several organic acids and SCFAs, which help to maintain the low pH in the intestinal lumen [88]. Probiotic supplementation improved the production and distribution of tight junction proteins such as occludin, claudin, and JAM-1 [87–89] and mucin production [90].

Antioxidant system is one of the major protective mechanisms of the host because free radicals are associated with several cellular damages and subsequent diseases. Several studies proved that the supplementation of probiotics improved the antioxidant status of the host [40,91]. The supplementation of probiotics alters the host physiology such as regulation of polyamines, ornithine decarboxylase enzyme activity [76, 92], thereby reducing the risk of development of CRC.

Studies revealed that probiotic microbes can suppress the cancer cell proliferation and induce the apoptosis, which was mainly attributed to the production of SCFAs [93, 94].

The discussed mechanisms are affected by several factors such as probiotic strain, concentration, viability, duration of the consumption, and supplementation of dilatory fibers like prebiotics. Thus, not all the probiotics strains exhibit anti-CRC activities, it is necessary to screen the potent strain for the development of a probiotic-based therapeutic agent to control or prevent the incidence of CRC.

## 4. Conclusion

The multigenus and multistrain probiotics (VSL#3 containing *B. breve, B. infantis, B. longum, L. acidophilus, L. bulgaricus, L. casei, L. plantarum*, and *S. thermophilus* along with balsalazide *in vivo* study [35]) and single-genus and multistrain probiotic (*L. acidophilus* ATCC 314 and *L. fermentum* NCIMB 5221 [29] *in vitro* and *in vivo* study), single-strain probiotics (*L. rhamnosus GG* or *L. acidophilus* [37]; *L. plantarum* [42]; *L. casei* BL23 [45] *in vivo* study) are some of the probiotic strains reported as adjuvant therapeutic agent to manage the CRC. Single-strain probiotic (*L. rhamnosus* GG along with guar gum fiber [52]) is reported as the promising adjuvant therapeutic agent to manage the CRC-related complications in CRC patients. Several studies evidenced that the probiotic (single genus and multispecies probiotics includes *L. rhamnosus* R0011 and *L. acidophilus* R0052 [62]; multigenus and multispecies probiotics includes *L. casei, L. acidophilus*, *L. lactis, B. bifidum*, *B. longum*, and *B. infantis* strains along with ω-3 fatty acid [53]; LactoLevure^®^, multigenus and multispecies probiotics includes *B. lactis, L. acidophilus*, *L. plantarum*, and *S. boulardii* [70]), synbiotic (Synbiotic Forte™, multigenus and multispecies probiotics includes *P. pentosaceus*, *L. mesenteroides, L. paracasei, L. plantarum*, and prebiotics such as inulin, pectin, *β*-glucan, and resistant starch [71]) intervention improved the health status of CRC patients after surgery. The beneficial impact of probiotic supplementation relay on the host physiology, disease severity, strain, dosage, duration of intervention, other food supplementations, etc. The probiotic supplements improved the immune system and intestinal integrity, increased the antimicrobial defense, and nullified the carcinogenic compounds in CRC patients. However, not all the probiotic interventions showed effective positive health effects in CRC patients. Further investigations are strongly recommended to reveal the exact mechanism and the potential of probiotics in CRC prevention.

## Figures and Tables

**Table 1:** Key results of *in vitro* studies on probiotics and colorectal cancer.

Experimental model	Supplements (probiotics)	Key results	References
LS513 cells	Live or inactive *L. acidophilus*, *L. casei* (1 × 10^6^–1 × 10^9^ CFU/mL), and 5-fluorouracil (5-FU, 100 *µ*g/ml)	Dose-dependent enhancement of apoptotic activity of 5-FU. Exposure of 10^8^ CFU/mL ↑ apoptotic efficiency (40%) ↑ activation of caspase-3 ↓ p21 expression	[22]

HCT-116 cells	CFS from *L. casei* and *L. rhamnosus* GG (25% v/v)	↓ cell invasion ↓ MMP-9 ↑ ZO-1	[23]

HGC-27, and DLD-1 cells	Live or heat-killed *L. paracasei* IMPC2.1 and *L. rhamnosus* GG (1 × 10^8^ CFU/ml)	Inhibited cell growth and induced apoptosis	[24]

CaCo-2 cells	CFS (5, 10, 20%) and bacterial extract (1, 5%) of *L. acidophilus* ATCC 4356 and *L. casei* ATCC 39392	↓ cell proliferation, migration, and invasion ↑ apoptosis	[25]

5-FU-resistant HT-29 and HCT-116 cells	*L. plantarum* CFS (10 *μ*g) and 5-FU (50 *μ*M)	↓ expression of CD44, CD133, CD166, and ALDH1 ↑ caspase-3 activity ↓ Wnt/*β*-catenin signaling ↓ size and formation of colonospheres	[26]

HT-29 cells	CFS or metabolites of *Propionibacterium freudenreichii* ITG P9 with TRAIL and ITG P9-mediated fermented milk with TRAIL	↑ proapoptotic gene expression ↓ antiapoptotic gene expression, TRAIL-R1/DR4, TRAIL-R2/DR5, and caspase-dependent proapoptotic activity	[27]

HT-29 cells	CFS and cells of *Lactobacillus* strains (*L. brevis* PM150, *L. plantarum* PM153, *L. brevis* PM177, *L. delbrueckii* subsp. *bulgarius* BCRC10696, *L. reu*teri BCRC14625, *L. salivarius* BCRC14759, and *L. johnsonii* BCRC17010)	↑ nitric oxide secretion ↑ proapoptosis ↑ lactate dehydrogenase and inhibited the growth of HT-29 cells	[28]

CaCo-2 cells	*L. acidophilus* ATCC 314 and *L. fermentum* NCIMB 5221	↓ cell proliferation ↑ apoptosis	[29]

HT-29, and Caco-2 cells	Methanolic extract of metabolites of *Pichia kudriavzevii* AS-12 (65 and 75 *μ*g/ml)	Cytotoxic to cancer cells ↑ apoptosis	[30]

HT-29 cells	CFS from *Saccharomyces cerevisiae*	↑ *PTEN*, *Caspas3* expression and ↓ *Bclxl* and *RelA* expression at 24 h posttreatment ↓ cell growth	[31]

Human colonic smooth muscle strips	CFS, live cells and microbial fractions of *L. acidophilus* HB56003, *S. thermophilus* HB5621, *E. faecalis* HB62001, and *B. longum* HB55020.	Inhibited the contractility of colonic smooth muscle strips	[32]

MMP-9: matrix metalloproteinase-9; ZO-1: zona occludens-1; CFS: cell-free supernatant; 5-FU: 5-fluorouracil; TRAIL; TNF-related apoptosis-inducing ligand.

**Table 2:** Effect of probiotic supplementation in CRC experimental animals.

Experimental model	Intervention	Duration of treatment	Key results	References
Apc^Min/+^ CRC mouse model	*L. acidophilus* ATCC 314, *L. fermentum* NCIMB 5221 (each 0.5 × 10^10^ CFU; total 1 × 10^10^ CFU/day)	12 weeks	↓ multiplicity of tumors ↓ *β*-catenin and Ki-67	[29]

Azoxymethane-mediated colonic neoplasm induced Sprague-Dawley rats	*B. lactis* (1 × 10^11^ CFU/g), and/or resistant starch (^*∗*^Hi-maize® 958 or Hi-maize® 260; 100 g/kg diet)	∼22 weeks	↓ incidence and development of colonic neoplasms The protective effects were observed to be higher in the synbiotic supplemented group	[33]

Trinitrobenzene sulfonic acid-mediated chronic colitis induced Sprague-Dawley rats	VSL#3 (*B. breve, B. infantis, B. longum, L. acidophilus, L. bulgaricus, L. casei, L. plantarum*, and *Streptococcus salivarius* subsp. *thermophilus*), 5 × 10^9^ CFU/100 g of body weight	Differs^*∗∗*^	No carcinoma development No high-grade dysplasia ↓ colon damage ↑ expression of antiangiogenic factor angiostatin, alkaline sphingomyelinase, and vitamin D receptor.	[34]

Azoxymethane/dextran sodium sulfate-mediated colitis-associated CRC induced mouse model	VSL#3 (1.3 × 10^6^ CFU/day), and/or Balsalazide (300 mg/kg body weight/day)	2 weeks before azoxymethane exposure and continued for 9 weeks until sacrification	↓ number of tumors ↓ F4/80-positive macrophages ↓ p-STAT3 expression ↓ BCL-2 expression ↓ MIP-1*β*, MCP-1, IL-6, IL-10 level ↑ BAX expression	[35]

Azoxymethane-mediated colitis-associated CRC induced mouse model	VSL#3 (1 × 10^9^ CFU/day)	19 weeks (from 6^th^ week to 24^th^ week)	↓ *Clostridium* species No reduction in tumorigenesis	[36]

1,2-Dimethyl hydrazine (DMH)-mediated CRC induced Sprague Dawley rats	*L. rhamnosus GG,* or *L. casei, L. plantarum,* or *L. acidophilus,* or *B. bifidum.* (probiotic dose: 1 × 10^9^ CFU/day)	Seven weeks (1 week before starting DMH exposure and continued for 6 weeks)	↓ percentage of Aberrant crypt foci (ACF) ↓ nitroreductase activity, *β*-glucuronidase activity, *β*-glucosidase activity	[37]

DMH-mediated CRC-induced Sprague Dawley rats	Synbiotic (*L. rhamnosus GG, L. acidophilus,* and inulin; 1 × 10^9^ CFU probiotic +5 mg inulin/day) or probiotic (*L. rhamnosus GG,* and/or *L. acidophilus*; 1 × 10^9^ CFU probiotic/day) or prebiotic (inulin; 5 mg/day)	19 weeks (1 week before starting DMH exposure and continued for 18 weeks)	↓ MDA level ↑ GSH, SOD, and GPx Improved the histopathological score	[38]

DMH-mediated CRC-induced rats	*L. acidophilus* LaVK2 and *B. bifidum* BbVK3 or both probiotic + piroxicam; 2 × 10^9^ CFU/g of each probiotic	32 weeks	↓ number of ACF, mucin-depleted foci, and proliferating cell nuclear antigen	[39]

DMH-mediated CRC-induced rats	*L. plantarum* AS1 (1 × 10^9^ CFU/day)	5–21 weeks	↑ antioxidant system of the host ↓ tumor diameter and number of tumors	[40]

DMH-mediated CRC-induced rats	*L. salivarius* (5 × 10^8^ or 1 × 10^10^ CFU/kg body weight/day)	15 weeks (2 week before starting DMH exposure and continued until 15 weeks)	Improved the colonic microflora and luminal metabolisms. ↓ number and multiplicity of ACF, azoreductase activity ↑ short-chain fatty acid levels	[41]

CT26 tumor-bearing mice	*L. plantarum* or *L. rhamnosus*; 1 × 10^9^ CFU/day	Pre-exposure for 14 days	↓ CT26 growth ↑ lifespan of tumor-bearing mice ↑ IFN-*γ*, Th1-type CD4^+^ T differentiation ↑ CD8^+^ function ↑ NK cell infiltration	[42]

DMH-mediated CRC-induced rats	*L. salivarius* Ren (5 × 10^10^ CFU/kg body weight/day)	32 weeks	Reversed the DMH-induced altered microbiota	[43]
DMH-mediated CRC-induced rats	*L. rhamnosus* GG CGMCC 1.2134 (1 × 10^9^ CFU/day)	25 weeks	↓ incidence, multiplicity, and volume of tumor ↓expression of inflammatory proteins, and antiapoptotic protein ↑ proapoptotic proteins	[44]

DMH-mediated CRC-induced mice	*L. casei* BL23 (10 *µ*l; 1 × 10^8^ CFU/*µ*l)	3 days (on days 0, 14, 28)	↓ incidence of tumor ↓ multiple plaque lesions Regulates the T_reg_ and Th17 T cells Altered the expression of IL-6, IL-10, IL-17, and TGF-*β*	[45]

DMH and SW480 cell-mediated CRC-induced rat	*B. infantis* (1 × 10^9^ CFU/day) and/or 5-FU + oxaliplatin	11 days	↑ body weight and intestinal villus height ↓ IL-6, IL-1*β*, TNF-α levels, and Th17 and Th1 cell-associated cytokines ↑ CD4^+^, CD25^+^, Foxp^3+^, T_regs_ expressions	[46]

Azoxymethane/dextran sodium sulfate-mediated colitis-associated cancer-induced mice model	*B. longum, L. acidophilus*, and *E. faecalis* (1.2 × 10^7^ CFU/day)	Pretreatment for 2 weeks and continued till the end of the experiment	↓ intestinal inflammation and tumor formation. ↓ *Desulfovibrio*, *Mucispirillum*, and *Odoribacter* species ↑ *Lactobacillus* species Altered the expression of CXCR2 ligand genes	[47]

Azoxymethane-mediated CRC-induced mice	*L. acidophilus* (1 × 10^9^ CFU/day) and *B. bifidum* (1 × 10^9^ CFU/day)	5 months	↓ miR-135b, miR-155, and KRAS ↑ miR-26b, miR-18a, APC, PU.1, and PTEN	[48]

Azoxymethane/dextran sodium sulfate-mediated CRC-induced rat model	*L. acidophilus* LA5 and/or *B. animalis* subsp. *Lactis* BB-12, and GBR; 5 × 10^7^ CFU of single probiotic strain/day or 2.5 × 10^7^ CFU each strain/day	10 weeks	↓ mucin-depleted foci formation ↑ expression of p53, Bax, caspase-3, and Bax/Bcl-2 ratio ↓ Bcl-2 expression ↑ SOD activity ↓ aberrant crypt foci (ACF)-producing sialomucin	[49]

DMH-mediated CRC-induced rats	*L. rhamnosus* GG MTCC #1408, and/or *L. acidophilus* NCDC #15 (1 × 10^9^ CFU/day), and/or celecoxib (6 mg/kg body weight)	18 weeks	↓ multiplicity and tumor burden ↓ Bcl-2, K-ras expression ↑ Bax, p53 expression	[50]

DMH-mediated CRC-induced rats	*L. rhamnosus* GG MTCC #1408, and/or *L. acidophilus* NCDC #15 (1 × 10^9^ CFU/day), and/or celecoxib (6 mg/kg body weight)	6 weeks	↓ ACF formation ↓ *β*-catenin, COX-2, and NF-*κ*B expression	[51]

↑: increased; ↓: decreased; MDA: malondialdehyde; GSH: glutathione; SOD: superoxide dismutase; GPx: glutathione peroxidase; NK: natural killer; IFN-*γ*: interferon-*γ*; p-STAT3: phospho-signal transducer and activator of transcription 3; BCL-2: B-cell lymphoma 2; BAX: BCL2-associated X protein; MIP-1*β*: macrophage inflammatory protein 1 beta; MCP-1: monocyte chemoattractant protein-1; IL-6: interleukin-6; IL-10: interleukin-10; KRAS: Kirsten rat sarcoma 2 viral oncogene homolog; GBR: germinated brown rice; ^*∗*^Hi-maize® (high-amylose maize starch was used as a source of resistant starch); ^*∗∗*^From one week before colitis induction to death of the experimental animal.

**Table 3:** Probiotic supplementation and CRC: outcomes of clinical studies on human subjects.

Subjects	Place of study	Intervention	Duration	Key results	References
CRC patients undergoing adjuvant chemotherapy; *n* = 150 (74 females, 76 males); age = 31 to 75 years	Helsinki University Central Hospital, Finland	*Lactobacillus rhamnosus* GG (1–2 × 10^10^/day) capsules, and guar gum fiber containing nutritional supplement	24 weeks	↑ abdominal comfort level ↓ stool frequency	[52]

CRC patients undergoing chemotherapy; *n* = 140; age = 18 years and above	University of Malaya Medical Centre, Malaysia	*L. casei, L. acidophilus*, *L. lactis, B. bifidum, B. longum, B. infantis* (30 × 10^9^ CFU/sachet; 2 sachets per day) and *ω*-3 fatty acid (2 g per day)	4 weeks of probiotics and 8 weeks of ω-3 fatty acid	Improved the quality of life and inflammatory status of the CRC patients	[53]

CRC and polypectomized patients; *n* = 80; age = 40 to 70 years	Mercy University Hospital, Cork, Ireland	*L. rhamnosus* GG, *B. lactis* Bb12 (1 × 10^10^ CFU of both probiotics in a capsugel), oligofructose-enriched inulin (12 g per sachet per day)	12 weeks	↑ *Lactobacillus* and *Bifidobacterium* species ↓ *Clostridium perfringens* ↓ colorectal proliferation ↑ barrier function and IFN-*γ* production ↓ genotoxins exposure and IL-2 secretion	[54]

CRC patients; *n* = 22 (10 females, 12 males); age = 40 to 75 years, and healthy volunteers; *n* = 11 (5 females, 6 males); age = 40 to 75 years	Sixth People's Hospital, Shanghai Jiao Tong University, Shanghai, China	*B. longum, L. acidophilus*, and *Enterococcus faecalis* (1 × 10^7^ CFU/g; 2 g per capsule; 6 × 10^7^ CFU/day)	5 days	↑ density and diversity of mucosal microbiota in CRC patients ↓ *Fusobacterium* species in CRC patients	[55]

Human volunteers^*∗*^; *n* = 380; age = 40 to 65 years	Osaka Medical Centre for Cancer and Cardiovascular Diseases, Osaka, Japan	*L. casei* strain Shirota (1 × 10^10^ CFU/g of powder/after each meal) or wheat bran biscuits (7.5 g wheat bran in 25 g biscuits per day) or both	4 years	The incidence of tumor formation was low in the probiotic group; prevented the atypia of colorectal tumors.	[56]

Healthy human subjects; *n* = 20 (7 females, 13 males); age = 21 to 75 years		*B. lactis* (1 × 10^9^ CFU/g; 5 g/capsule/day) and/or resistant starch (12.5 g per sachet; 25 g per day)	Each intervention lasts for 4 weeks with no washout period^#^	↑ *Lachnospiraceae* spp. level No changes in serum biomarkers and epithelial proliferation	[57]

CRC patients^*∗∗*^; *n* = 31 (9 females, 22 males); age = 18 to 80 years	Department of Surgery, San Gerardo Hospital, Milano-Bicocca University, Monza, and Department of Surgery, San Raffaele Hospital, Vita e Salute University, Milan, Italy	*B. longum* and *L. johnsonii* (2 × 10^7^ or 2 × 10^9^ CFU per day); the powdered form of probiotics were consumed by mixing in nutritional supplement (100 mL)	3 days before surgery, on the day of surgery, and 2 days after surgery	*L. johnsonii* was observed in patients' fecal samples but not *B. longum.* Adhesion of *L. johnsonii* was directly correlated with the probiotic dose ↓ *Enterobacteriaceae* count in high-dose probiotic group ↑ CD3, CD4, CD8 and lymphocyte subsets expression Dendritic cells were not activated and affected	[58]
CRC patients^*∗∗*^; *n* = 100 (41 females, 59 males); age = 45 to 75 years	Sixth People's Hospital, Shanghai Jiao Tong University, Shanghai, China	*L. plantarum* L. *acidophilus B. longum* (2 g of encapsulated probiotics containing total 2.6 × 10^14^ CFU/day)	6 days before surgery and 10 days after surgery	↑ transepithelial resistance ↓ lactulose/mannitol ratio ↓ transmucosal permeability ↓ bacterial translocation ↑ tight junction protein expression ↓ enteropathogenic bacteria load ↓ incidence of diarrhea and infections	[59]

CRC patients^*∗∗*^; *n* = 150 (72 females, 78 males); age = 45 to 75 years	Sixth People's Hospital, Shanghai Jiao Tong University, Shanghai, and Sixth Affiliated Hospital of Sun Yat-sen University, Guangzhou, China	*L. plantarum* L. *acidophilus B. longum* (2 g of encapsulated probiotics containing total 2.6 × 10^14^ CFU/day)	6 days before surgery and 10 days after surgery	↓ serum zonulin ↓ duration of postoperative pyrexia, infection, and antibiotic treatment ↓ p38 MAPK pathway	[60]

CRC patients; *n* = 15 (9 females, 6 males); age = 68 to 75 years. Noncancer control group (individuals with normal colonic mucosa); *n* = 21 (17 females, 4 males); age = 55 to 73 years.		2 tablets containing 1.4 × 10^10^ CFU of *B. lactis,* 7 × 10^9^ CFU of *L. acidophilus*, inulin (630 mg) per day	8–78 days	Improved the diversity and abundance of butyrate-producing bacteria (*Clostridiales* and *Faecalibacterium* species) in fecal and mucosal microbiota of CRC patients Significant reduction of *Fusobacterium* and *Peptostreptococcus* species in fecal microbiota of CRC patients	[61]

CRC survivors^##^; *n* = 60 (25 females, 35 males); age = 45 to 70 years	Clinical Trial Centre in Severance hospital, Yonsei University, Republic of Korea	*L. rhamnosus* R0011, and *L. acidophilus* R0052 (2 × 10^9^ CFU/tablet, twice a day)	12 weeks	↓ irritable bowel symptoms Improved the overall quality of the life	[62]

CRC patients^*∗∗*^; *n* = 156 (65 females, 91 males); age = 45 to 75 years	Fukuoka University Hospital, Fukuoka, Japan	BIO-THREE®(2 mg of *E. faecalis* T110, 10 mg of *Clostridium butyricum* TO-A, and 10 mg of *Bacillus mesentericus* TO-A per tablet); 6 tablets per day	3–15 days before surgery	↓ superficial incisional surgical site infections Improved the microbiota	[63]

CRC patients^*∗∗*^; *n* = 40 (16 females, 24 males); age = 45 to 80 years		*L. acidophilus, L. casei, L. lactis, B. infantis. B. bifidum,* and *B. longum* (30 × 10^9^ CFU/sachet; twice per day)	7 days before surgery	Improved the gut function, and reduced the duration of hospital stay after surgery	[64]

CRC patients^*∗∗*^; *n* = 60 (33 females, 27 males); age = 45 to 80 years	Sixth People's Hospital, Shanghai Jiao Tong University, Shanghai, China	Probiotic powder containing *B. longum* (1 × 10^7^ CFU/g)*, L. acidophilus* (1 × 10^7^ CFU/g) and *E. faecalis* (1 × 10^7^ CFU/g)	5 days before surgery and 7 days after surgery	Improved the bowel function ↓ incidence of diarrhea	[65]
CRC patients^*∗∗*^; *n* = 164 (49 females, 115 males); age = 45 to 80 years	Department of Surgery of the AHEPA University Hospital of Thessaloniki, Greece	1.75 × 10^9^ CFU of *B. lactis* BB-12, 1.75 × 10^9^ CFU of *L. acidophilus* LA-5, 0.5 × 10^9^ CFU of *L. plantarum,* and 1.5 × 10^9^ CFU of *S. boulardii* per capsule; twice per day	16 days (1 day before surgery and 15 days after surgery) and 30 days of follow-up period	↓ anastomotic leakage, pneumonia, and infection in surgical site. Altered the expression of IL-6, TNF, and SOCS3	[70]

CRC patients^*∗∗*^; *n* = 75 (32 females, 43 males); age = 60 to 75 years	First Department of Propaedeutic Surgery of Athens Medical School at Hippocration Hospital, Athens, Greece	Each 12 g of synbiotic sachet contains probiotics (*Pediococcus pentosaceus, Leuconostoc mesenteroides, L. paracasei, L. plantarum*; each 10 × 10^11^ CFU) and prebiotics (inulin, resistant starch, pectin, and b-glucan; each 2.5 g); 1 sachet per day	15 days	↑ gastrointestinal Quality of Life Index Improved the functional bowel disorder score	[71]

↑: increased; ↓: decreased; IFN-*γ*: interferon-*γ*; IL-2: interleukin-2; IL-6: interleukin-6; CRC: colorectal cancer; p38 MAPK: p38 mitogen-activated protein kinase; TNF: tumor necrosis factor; SOCS3: suppressor of cytokine signaling 3. ^*∗*^Patients who had surgical elimination of at least 2 colorectal tumors; ^*∗∗*^Patients undergoing colorectal surgery; ^#^Intervention of probiotic, prebiotics, and synbiotics in a sequential way, and each intervention last for 4 weeks;^##^CRC patients those who have completed their treatment for the disease.
